# Long Noncoding RNA lncRNA-3 Recruits PRC2 for MyoD1 Silencing to Suppress Muscle Regeneration During Aging

**DOI:** 10.3390/ijms252212478

**Published:** 2024-11-20

**Authors:** Zong-Kang Zhang, Daogang Guan, Jintao Xu, Xiaofang Li, Ning Zhang, Shanshan Yao, Ge Zhang, Bao-Ting Zhang

**Affiliations:** 1 School of Chinese Medicine, Faculty of Medicine, The Chinese University of Hong Kong, Hong Kong SAR, China; maxzhangzk@cuhk.edu.hk (Z.-K.Z.);; 2 Law Sau Fai Institute for Advancing Translational Medicine in Bone and Joint Diseases, School of Chinese Medicine, Hong Kong Baptist University, Hong Kong SAR, China

**Keywords:** long noncoding RNA, polycomb repressive complex 2, *MyoD1*, RbAp46/48, muscle regeneration

## Abstract

Lowered muscle regenerative capacity in the elderly greatly contributes to the development of multiple diseases. The specific roles of long noncoding RNAs (lncRNAs) in muscle regenerative capacity during aging remain unknown. Here, we identify an elevated lncRNA (lncRNA-3), in association with reduced MyoD expression and suppressed muscle regenerative capacity, in the skeletal muscle of aged mice. LncRNA-3 could interact with both the *MyoD1* promoter and RbAp46/48, a subunit of Polycomb repressive complex 2 (PRC2). LncRNA-3 could recruit PRC2 to the *MyoD1* promoter and enhance the *MyoD1* silencing, which, in turn, suppressed the muscle regenerative capacity. Muscle-specific lncRNA-3 knockdown could restore the muscle regenerative capacity in the aged mice. Exogenous RbAp46/48 binding motif (Rb-motif-2) treatment in skeletal muscle could compete for the lncRNA-3 binding, and therefore, enhance the muscle regenerative capacity in the aged mice. Taken together, lncRNA-3 requires PRC2 for *MyoD1* silencing to suppress muscle regenerative capacity during aging. These findings provide a novel therapeutic target and a new strategy to elevate the muscle regenerative capacity in the aged population.

## 1. Introduction

Skeletal muscle is one of the largest organs in the human body; it plays a critical role in voluntary movement and has several other functions, such as metabolic and endocrine functions [1]. Skeletal muscle maintains its function and size through regeneration after muscle injury. Satellite cells play the dominant roles in muscle homeostasis and regeneration [2]. Satellite cells are activated in response to both physiological stimuli (e.g., exercise) and under pathological conditions (e.g., injury and degenerative diseases) to generate myoblasts that are capable of fusion and differentiation [3]. Even after severe and repetitive muscle injuries, appropriate regeneration can recover muscle function.

The world’s population is aging rapidly. Aging is accompanied by a progressive decline in tissue function and increased vulnerability to disease. The current understanding of the aging process centers on the interplay of cell-intrinsic intercellular communication and systemic dysregulations. The muscle regenerative capacity is lowered in the skeletal muscle of the elderly [4,5]. The imbalance between muscle injury and regeneration causes the deterioration of muscle function, resulting in the development of multiple diseases. Thus, it is highly desirable to explore the molecular understandings of lowered muscle regenerative capacity in the elderly for developing an alternative muscle anabolic strategy.

Long noncoding RNAs (lncRNAs) are non-protein-coding RNAs with lengths above 200 nucleotides, but they perform various crucial functions within the cell [6]. Over the years, many researchers unveiled the association between lncRNAs and skeletal muscle differentiation and regeneration, including Synaptopodin-2 (SYNPO2) intron sense-overlapping lncRNA (SYISL) [7], PRKG1-AS1 [8], Malat1 [9] and lnc-mg [10]. A recent study reviewed the molecular functions of lncRNAs in age-related muscle pathology and suggested that lncRNAs might be promising therapeutic targets for muscle aging [11].

Polycomb repressive complex 2 (PRC2) is a histone methyltransferase that methylates histone H3 at lysine 27 (H3K27) [12]. High levels of H3K27 trimethylation (H3K27me3) in the coding region generally correlate with transcription repression [13]. The core PRC2 complex, which is conserved from *Drosophila* to mammals, comprises four components: Enhancer of zeste (EZH2), Suppressor of zeste 12 (SUZ12), Extra-sex combs (EED) and Nurf55 (RbAp46/48) [14]. Recent reports have shown that lncRNAs are associated with gene silencing through guiding PRC2 to the histones in target genes [15,16]. However, the role of lncRNA in modulating the posttranslational modification of histone during aging remains unclear.

We recently identified a novel lncRNA (lncRNA-3) that is negatively associated with the muscle mass in aged mice. We revealed that lncRNA-3 could guide histone methyltransferase PRC2 to the *MyoD1* promoter to mediate gene silencing, thereby suppressing the muscle regenerative capacity. In addition, lncRNA-3 knockdown and exogenous target protein binding motif treatment in skeletal muscle can enhance the muscle regenerative capacity in aged mice.

## 2. Results

### 2.1. Elevated lncRNA-3 Expression Level in Skeletal Muscle Was Accompanied by Decreased Muscle Mass and Reduced Muscle Regenerative Capacity in Aged Mice

**Figure 1:**
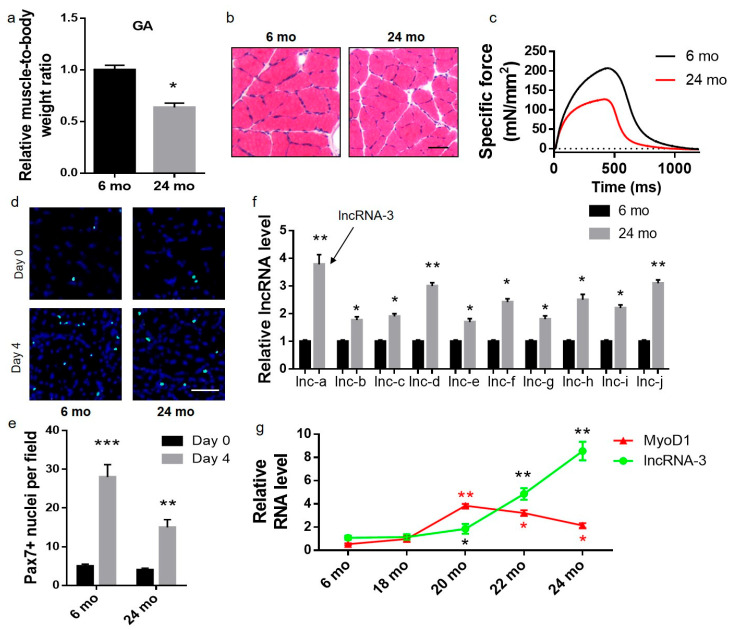
Elevated lncRNA-3 expression level in the skeletal muscle was accompanied by a decreased muscle mass and reduced muscle regenerative capacity in the aged mice. (**a**–**c**) Gastrocnemius (GA) muscle-to-body weight ratio (**a**), H&E staining of the cross-section of GA muscle (**b**) and the GA muscle specific force (**c**) between the adult and aged mice. (**d**,**e**) Representative images (**d**) and quantification analysis (**e**) of the gastrocnemius muscle cross-sections from adult and aged mice on day 0 and day 4 post-muscle injury stained for Pax7 (green) and nuclei (blue). Scale bar: 50 µm. (**f**) Real-time PCR analysis of top 10 upregulated lncRNA levels in the GA muscle of the adult and aged mice. The most upregulated one was named lncRNA-3. (**g**) Relative expression levels of lncRNA-3 (green) and the *MyoD1* mRNA (red) in the GA muscle from the mice at different ages. GAPDH and U6 were used as endogenous controls. *n* = 10. Data are presented as the mean ± SEM. * *p* < 0.05, ** *p* < 0.01 vs. 6 mo. (**e**) ** *p* < 0.01, *** *p* < 0.001 vs. day 0.

Comparing the skeletal muscle mass between the aged and adult mice, the muscle mass in the aged mice was significantly lower than that in the adult mice, and the gastrocnemius muscle atrophied the most significantly (Figure 1a). Similar muscle atrophy was also observed in other skeletal muscles of the aged mice, including the tibialis anterior (TA) and soleus muscles (Supplementary Figure S1). Consistently, the muscle fiber size and the muscle strength in the gastrocnemius muscle of the aged mice were also dramatically lower than those in the adult mice (Figure 1b,c). In the cardiotoxin (CTX)-induced skeletal muscle injury animal model, the satellite cell proliferation in the muscle following the injury was significantly lower in the aged mice than that in the adult mice (Figure 1d,e). The significantly upregulated lncRNAs in the skeletal muscle of the aged mice compared with the adult mice were identified by RNA sequencing (Supplementary Table S1). Real-time polymerase chain reaction (PCR) analysis confirmed that the expression levels of the candidate lncRNAs were upregulated in the skeletal muscle of the aged mice (Figure 1f). The most upregulated lncRNA in the skeletal muscle was a 568 nt transcript derived from a gene located on chromosome 2 of the mouse genome (NR_108091.1), which is named lncRNA-3 in the current paper (Supplementary Figure S2). The expression level of lncRNA-3 was stable at a modest level from ages 6 months to 18 months, then started to elevate moderately from ages 18 months to 20 months (early stage of aging) and increased dramatically from ages 20 months to 24 months (advanced aging). MyoD was stable at a low level from ages 6 months to 18 months, then started to increase from ages 18 months to 20 months (early stage of aging) and decreased from ages 20 months to 24 months (advanced aging). The lncRNA-3 level and MyoD expression were negatively correlated during advanced aging (Figure 1g). Similarly, the expression level of lncRNA-3 was higher in the TA and soleus muscles of the aged mice than those of the adult mice (Supplementary Figure S1).

### 2.2. LncRNA-3 Inhibited Myogenic Differentiation of Mouse Muscle Satellite Cells In Vitro and Suppressed Skeletal Muscle Regeneration in Adult Mice 

**Figure 2:**
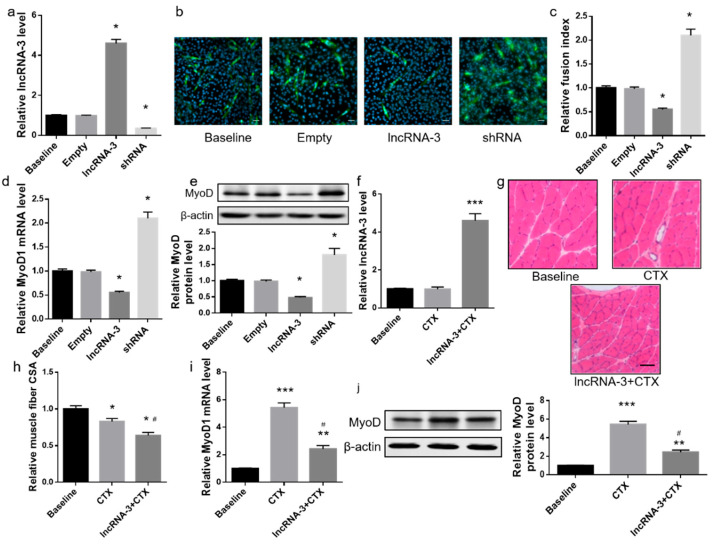
LncRNA-3 inhibited the myogenic differentiation in vitro and muscle regenerative capacity in mice. (**a**) Expression levels of lncRNA-3 of mouse muscle satellite cells transduced with either lncRNA-3 or shRNA vectors on day 7 of differentiation. (**b**) Representative images of mouse muscle satellite cells transduced with either lncRNA-3 or shRNA vectors on day 7 of differentiation. Myosin was labeled with green fluorescence, and the nuclei were labeled with DAPI. Scale bar: 50 μm. (**c**) The fusion index determined by the number of nuclei within the myosin-positive myotubes in each group on day 7 of differentiation. (**d**,**e**) Expression levels of the *MyoD1* mRNA (**g**) and MyoD protein (**h**) in the mouse muscle satellite cells in each group on day 7 of differentiation. (**f**) Real-time PCR analysis of the lncRNA-3 level in the gastrocnemius muscle of empty- or lncRNA-3-vector-infected mice following the CTX treatment. (**g**–**j**) Cross-sections from the mid-belly gastrocnemius muscle (**g**), muscle fiber cross-sectional area (CSA) (**h**), expression levels of the *MyoD1* mRNA (**i**) and MyoD protein (**j**) in the gastrocnemius muscle of empty- or lncRNA-3-vector-infected mice 28 days after the CTX treatment. Scale bar: 50 µm. Data are presented as the mean ± SEM. *n* = 3 for in vitro. *n* = 10 for in vivo. * *p* < 0.05, ** *p* < 0.01, *** *p* < 0.001 vs. baseline. ^#^ *p* < 0.05 vs. CTX.

To further explore the biological functions of lncRNA-3 in muscle satellite cells, we constructed an adeno-associated virus (AAV) vector that encoded either lncRNA-3 or lncRNA-3 shRNA (Supplementary Figure S3). LncRNA-3 knockdown in muscle satellite cells significantly enhanced the mRNA and protein levels of the myogenic markers (MyoD). Moreover, the lncRNA-3 knockdown enhanced the satellite cell differentiation and myotube formation. Meanwhile, the lncRNA-3 overexpression decreased the above in the muscle satellite cells (Figure 2a–e). We further overexpressed the lncRNA-3 level in the adult mice by an intramuscular injection of a muscle-specific AAV vector constructed with full-length lncRNA-3. The skeletal muscle injury model was then established by an intramuscular injection with CTX in the gastrocnemius muscles of the adult mice. Enforced lncRNA-3 expression in the mice delayed the skeletal muscle regeneration process following injury (Figure 2f–h). Moreover, the lncRNA-3 overexpression inhibited the MyoD expression (Figure 2i,j). These data indicate that lncRNA-3 could inhibit the MyoD expression in muscle satellite cells and suppress the muscle regenerative capacity in mice. 

### 2.3. LncRNA-3 Interacted with Histone Methyltransferase PRC2 and Mediated MyoD1 Gene Silencing In Vitro

**Figure 3:**
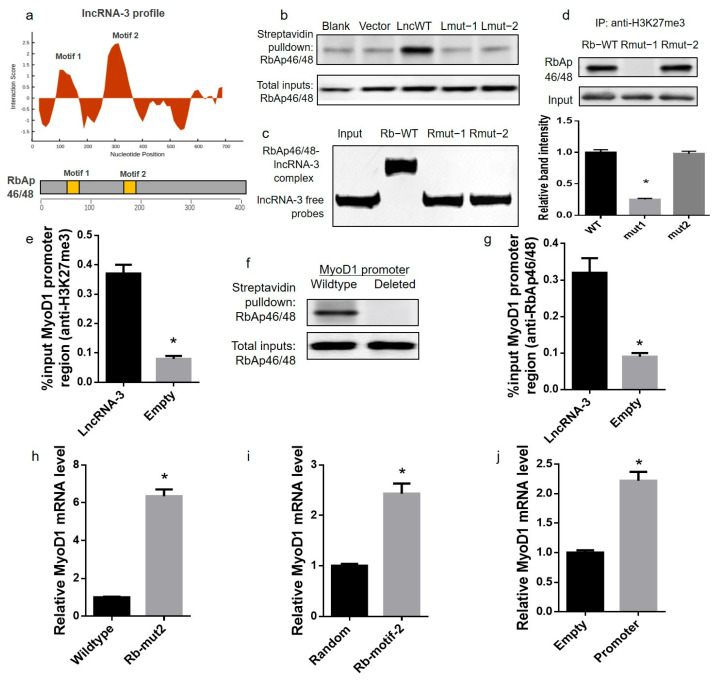
LncRNA-3 interacted with the histone methyltransferase PRC2 and mediated the *MyoD1* gene silencing in vitro. (**a**) Schematic of the interaction propensity for the full-length lncRNA-3 with RbAp46/48, as predicted by CatRAPID. (**b**) Representative Western blot analysis of the tagged-RNA streptavidin pulldown assay. (**c**) Representative RNA EMSA analysis of the flag-tagged RbAp46/48 pulldown assay. (**d**) Representative Western blot analysis of the RbAp46/48 co-immunoprecipitation assay using the anti-H3K27me3 antibody. (**e**) Chromatin immunoprecipitation (ChIP) assay using the anti-H3K27me3 antibody in the lncRNA-3- or empty-vector-transduced mouse muscle satellite cells, where the %input of the *MyoD1* promoter was detected by real-time PCR. (**f**) Representative Western blot analysis of the tagged-RNA streptavidin pulldown assay. Full-length or deletion fragments of the *MyoD1* promoter were transfected into the lncRNA-3-vector-transduced mouse muscle satellite cells and followed with the tagged-DNA streptavidin pulldown assay. (**g**) ChIP assay using anti-RbAp46/48 antibody in the lncRNA-3- or empty-vector-transduced mouse muscle satellite cells, where the %input of the *MyoD1* promoter was detected by real-time PCR. (**h**) Real-time PCR analysis of the *MyoD1* mRNA level in the mouse muscle satellite cells transfected with the wildtype or mutated RbAp46/48 (Rmut2). (**i**) Real-time PCR analysis of the *MyoD1* mRNA level in the mouse muscle satellite cells transduced with lncRNA-3 and treated with peptide with a random sequence (random) or RbAp46/48 motif 2 (Rb-motif-2). (**j**) Real-time PCR analysis of the *MyoD1* mRNA level in mouse muscle satellite cells transduced with lncRNA-3 and treated with the *MyoD1* promoter overexpression vector and empty vector. Data are presented as the mean ± SEM. *n* = 3. (**d**) * *p* < 0.05 vs. Rb-WT. (**e**,**g**) * *p* < 0.05 vs. lncRNA-3 vector. (**h**) * *p* < 0.05 vs. wildtype. (**i**) * *p* < 0.05 vs. random.

To determine the potential target proteins of lncRNA-3 in the mouse muscle satellite cells in vitro, in vitro transcribed biotinylated (Bi)-lncRNA-3 (Bi-Lnc3) or Bi-Luciferase (Bi-LUC) RNA was incubated with the lysis of mouse muscle satellite cells and followed by streptavidin pulldowns. The mass spectrometry was performed for the enriched proteins. The PRC2 subunit RbAp46/48 was found to be one of the top 20 enriched proteins in the Bi-Lnc3-transcribed mouse muscle satellite cells (Supplementary Table S2). By a bioinformatics analysis, it was further predicted that the binding between RbAp46/48 and lncRNA-3 depended on the two motifs on lncRNA-3 (Lnc-motif 1: no. 126–177 nucleotide; Lnc-motif 2: no. 313–364 nucleotide) and two motifs on RbAp46/48 (Rb-motif 1: Pro50-Ala61; Rb-motif 2: Asn185-His191) (Figure 3a). Based on the prediction data, either the biotinylated full-length (WT) or two mutated lncRNA-3, including the truncation of motif 1 (Lnc-mut1) and motif 2 (Lnc-mut2), was synthesized and transfected into mouse muscle satellite cells and followed with a tagged-RNA streptavidin pulldown assay. RbAp46/48 was only enriched in the mouse muscle satellite cells transfected with the WT lncRNA-3 (Figure 3b), suggesting that motifs 1 and 2 of lncRNA-3 participated in the interaction between lncRNA-3 and RbAp46/48. Moreover, we designed and synthesized two mutated RbAp46/48, including mutations at motif 1 (Rb-mut1) and motif 2 (Rb-mut2) of RbAp46/48. The flag-tagged wildtype or two mutated RbAp46/48 were transfected into mouse muscle satellite cells and followed with RNA immunoprecipitation (RIP). The RNA electrophoretic mobility shift assay (EMSA) demonstrated that lncRNA-3 was only enriched in the mouse muscle satellite cells transfected with wildtype RbAp46/48, suggesting that motifs 1 and 2 of RbAp46/48 participated in the interaction between lncRNA-3 and RbAp46/48 (Figure 3c). To determine the roles of the two binding sites of RbAp46/48 (motifs 1 and 2) to lncRNA-3 in regulating the PRC2 function as a histone methyltransferase, the enrichment of the mutated RbAp46/48 (Rb-mut1 and Rb-mut2) was evaluated in the mouse muscle satellite cells by co-immunoprecipitation (Co-IP) using the H3K27me3 (trimethylated form of the PRC2 target H3K27) antibody. The enrichment of Rb-mut2 was detected by Western blotting (Figure 3d), indicating that the mutation at motif 2 of RbAp46/48 could block the interaction between lncRNA-3 and RbAp46/48 without influencing the function of PRC2 as a histone methyltransferase.

To further determine the potential target gene locus of the lncRNA-3-PRC2 complex, a bioinformatics analysis was performed by CatRAPID. Among the potential target genes, real-time PCR analysis revealed that the mRNA level of *MyoD1* was the most upregulated in the lncRNA-3 shRNA-transfected mouse muscle satellite cells (Supplementary Figure S4). The enrichment of the *MyoD1* promoter was observed in the lncRNA-3-overexpressed mouse muscle satellite cells by chromatin immunoprecipitation (ChIP) using the anti-H3K27me3 antibody, indicating the lncRNA-3 overexpression could enhance the trimethylation level of H3K27 in the *MyoD1* promoter (Figure 3e). The interaction between the *MyoD1* promoter and lncRNA-3-PRC2 was further confirmed, as evidenced by the following: (1) the enrichment of RbAp46/48 was observed in the lncRNA-3-overexpressed mouse muscle satellite cells co-transfected with the full-length *MyoD1* promoter, but not with the deletion fragments of the *MyoD1* promoter (Figure 3f), and (2) the *MyoD1* promoter was more significantly enriched in the lncRNA-3-overexpressed mouse muscle satellite cells than in the empty-vector-transfected cells by ChIP analysis using the anti-RbAp46/48 antibody (Figure 3g).

To investigate whether lncRNA-3 required RbAp46/48 in PRC2 to enhance the *MyoD1* gene silencing during the muscle differentiation in vitro, lncRNA-3 was overexpressed in the mouse muscle satellite cells and the cells were transfected with wildtype or mutated RbAp46/48 (Rb-mut2). The mRNA expression level of *MyoD1* was significantly inhibited in the wildtype RbAp46/48-transfected mouse muscle satellite cells, but not in the mutated RbAp46/48-transfected mouse muscle satellite cells (Figure 3h). Furthermore, to investigate whether the blockage of the interactions between the lncRNA-3, RbAp46/48 and *MyoD1* promoter could inhibit lncRNA-3′s biological function, the lncRNA-3-overexpressed mouse muscle satellite cells were treated with a synthesized peptide that comprised RbAp46/48 binding motif 2 (Rb-motif-2), a *MyoD1* promoter overexpression system and corresponding random sequence controls. The *MyoD1* mRNA level was significantly higher in the Rb-motif-2 treatment group and *MyoD1* promoter overexpression group compared with the corresponding control groups (Figure 3i,j). Taken together, lncRNA-3 recruited PRC2 to the *MyoD1* promoter and enhanced *MyoD1* silencing during the muscle satellite cells proliferation and differentiation in vitro.

### 2.4. Skeletal-Muscle-Specific lncRNA-3 Knockdown, Rb-motif-2 Treatment and MyoD1 Promoter Overexpression Could Rescue Muscle Regenerative Capacity in Muscle-Specific lncRNA-3 Knockin Mice

**Figure 4:**
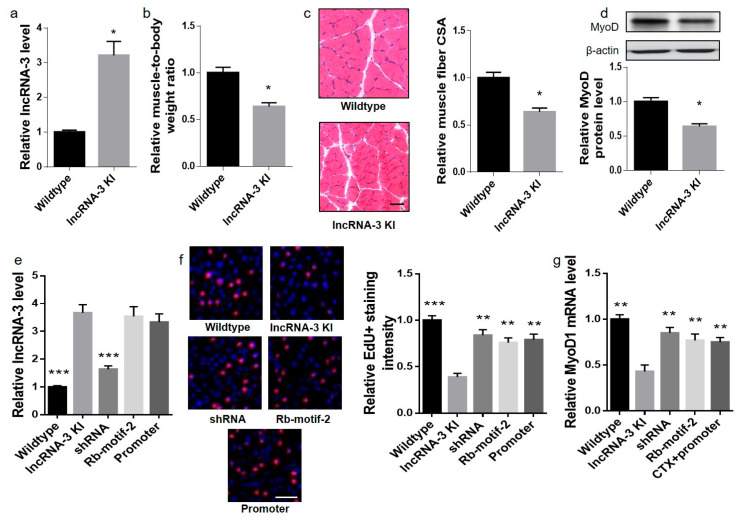
Skeletal-muscle-specific knockdown of lncRNA-3, the Rb-motif-2 treatment and the *MyoD1* promoter expression could rescue the muscle regenerative capacity in the muscle-specific lncRNA-3 knockin (Sk-lncRNA-3 KI) mice. (**a**) Real-time PCR analysis of the lncRNA-3 levels in the gastrocnemius muscle of the wildtype and Sk-lncRNA-3 KI mice. (**b**) Gastrocnemius muscle-to-body weight ratio in the wildtype and transgenic mice. (**c**) Cross-sections from the mid-belly gastrocnemius muscle (left) and muscle fiber CSA (right). Scale bar: 50 µm. (**d**) Western blot analysis of the MyoD protein level in each group. (**e**–**g**) Adult Sk-lncRNA-3 KI mice and wildtype mice were intramuscularly injected with CTX to induce muscle injury. The Sk-lncRNA-3 KI mice were followed with an intramuscular administration of lncRNA-3 shRNA, the synthesized peptide Rb-motif-2 and the *MyoD1*-promoter-overexpression system. (**e**) Real-time PCR analysis of the lncRNA-3 levels in the gastrocnemius muscle on day 7 post-treatment in each group. (**f**) Representative images (left) and the intensity (right) of EdU positive staining on day 7 post-treatment in each group. Red: EdU staining, blue: DAPI. (**g**) Real-time PCR analysis of the *MyoD1* mRNA levels on day 7 post-treatment in each group. *n* = 10. GAPDH and U6 small nuclear RNA were used as the endogenous controls of mRNA and lncRNA, respectively. β-actin was used as the endogenous control for MyoD protein. Data are presented as the mean ± SD. (**a**–**d**) * *p* < 0.05 vs. wildtype. (**e**–**g**) * *p* < 0.05, ** *p* < 0.01, *** *p* < 0.001 vs. lncRNA-3 KI.

To facilitate the mechanistic study of the functional role of lncRNA-3 in vivo, a skeletal-muscle-specific lncRNA-3 knockin (Sk-lncRNA-3 KI) mice was generated. ROSA26-PCAG-STOP^flox^–lncRNA-3 KI mice that carried the STOP^flox^-lncRNA-3 in the ROSA26 allele were successfully generated. Then, these mice were intercrossed with ACTA1-Cre mice to obtain the Sk-lncRNA-3 KI mice. The Sk-lncRNA-3 KI mice showed lower muscle mass, lower muscle fiber CSA and lower MyoD protein level in the gastrocnemius muscle compared with the wild-type mice (Figure 4a–d). Furthermore, the Sk-lncRNA-3 KI mice were injured at the gastrocnemius muscle by an intramuscular CTX injection, followed by an intramuscular injection of the lncRNA-3 shRNA system, Rb-motif-2 and *MyoD1* promoter overexpression system. The muscle satellite cell proliferation and *MyoD1* mRNA level were significantly lower in the Sk-lncRNA-3 KI mice than the wild-type mice after the intramuscular CTX injection. The lncRNA-3 knockdown, exogenous RbAp46/48 binding motif and *MyoD1* promoter overexpression could significantly promote the satellite cell proliferation and *MyoD1* mRNA level in the injured muscle of the Sk-lncRNA-3 KI mice (Figure 4e–g), indicating lncRNA-3 recruited PRC2 to the *MyoD1* promoter and enhanced *MyoD1* silencing and suppressed the muscle regenerative capacity in vivo.

### 2.5. Skeletal-Muscle-Specific lncRNA-3 Knockdown and Exogenous Rb-motif-2 Treatment Restored Muscle Regenerative Capacity in Aged Mice

**Figure 5:**
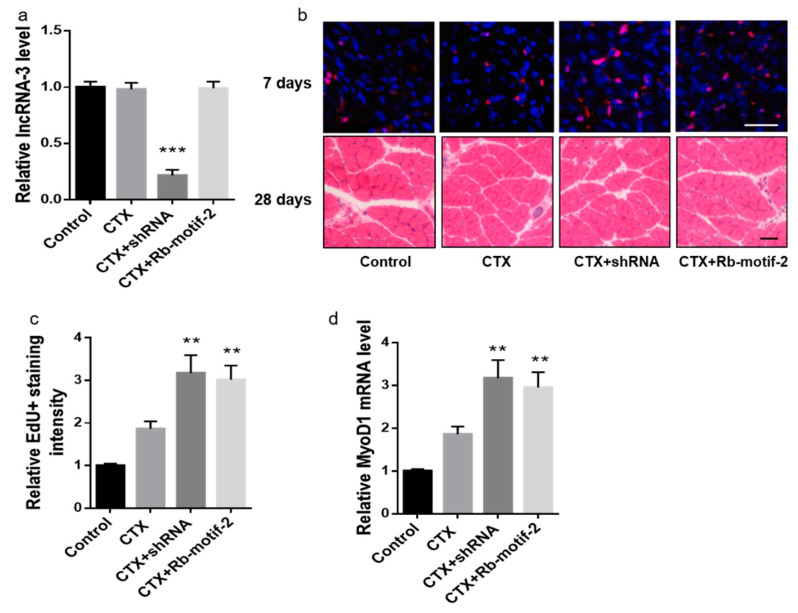
Skeletal-muscle-specific lncRNA-3 knockdown and exogenous Rb-motif-2 treatment restored the muscle regenerative capacity in the aged mice. (**a**) LncRNA-3 level in the gastrocnemius muscle of the aged mice treated with the lncRNA-3 shRNA system or exogenous Rb-motif-2 in the skeletal muscle following a muscle injury. (**b**) EdU staining (red) and the nuclei (blue) indicating muscle satellite cell proliferation at 7 days post-muscle injury (upper panel) and H&E staining of the cross-sections from the mid-belly gastrocnemius muscle at 28 days post-muscle injury (bottom panel). Scale bar: 50 µm. (**c**) The intensity of the EdU positive staining in each group at 7 days post-muscle injury. (**d**) Real-time PCR analysis of the *MyoD1* mRNA level in the gastrocnemius muscle at 7 days post-muscle injury. *n* = 10 for each group. Each sample was assessed in triplicate. GAPDH was used as the control for mRNA. Data are presented as the mean ± SEM. ** *p* < 0.01, *** *p* < 0.001 vs. CTX.

To investigate the effects of the lncRNA-3 knockdown and exogenous Rb-motif-2 treatment on the muscle regenerative capacity in the aged mice, 22-month-old mice were locally injected with either the lncRNA-3 shRNA system or exogenous Rb-motif-2, respectively, into the gastrocnemius muscle once and followed by a muscle injury induced by a CTX injection. The lncRNA-3 knockdown and exogenous Rb-motif-2 treatments restored the muscle regenerative capacity in the aged mice, as evidenced by the enhanced satellite cell proliferation, promoted muscle fiber CSA and increased *MyoD1* mRNA levels in the respective treatment group (Figure 5a–d).

## 3. Discussion

In this study, we identified lncRNA-3 in mice skeletal muscle whose expression was elevated during aging. The lncRNA-3 could recruit PRC2 to the *MyoD1* promoter to silence *MyoD1* expression and compromise the muscle regenerative capacity during aging, which revealed a paradigm of lncRNA-mediated epigenetic regulation in skeletal muscle regeneration during aging.

Emerging studies have reported that lncRNAs play important roles in muscle homeostasis, including H19 [17,18], linc-MD1 [19], Yam-1 [20], Malat1 [9] and lnc-mg [10], which were demonstrated to regulate muscle differentiation and regeneration. Satellite cells play the dominant role in muscle regeneration [2], which are able to fuse with existing myofibers, repairing damaged muscle fibers or alternatively fuse to each other to form new myofibers. Muscle regenerative capacity is compromised in the elderly [4,5]. The improvement in resistance to injury in skeletal muscle by regular resistance training occurs more slowly in the skeletal muscle of elderly humans/animals compared with young humans/animals [21,22,23]. In the current study, we observed that the elevated lncRNA-3 level in the aged skeletal muscle was associated with the compromised muscle regenerative capacity in the aged mice. By performing a series of in vitro and in vivo studies, we found that lncRNA-3 could inhibit the *MyoD1* expression and suppress the muscle regenerative capacity.

PRC2 is a key mediator of epigenetic regulation during the whole life span [24], where it methylates H3K27 and correlates with gene silencing [12]. There have been reports that lncRNAs are associated with gene silencing through guiding PRC2 to the histones in target genes [15,16]. In the current study, we not only demonstrated that lncRNA-3 could interact with PRC2 through directly binding with its component, RbAp46/48, but also confirmed that lncRNA-3 could target the *MyoD1* promoter. LncRNA-3 could interact with RbAp46/48 and recruit PRC2 to the *MyoD1* promoter for *MyoD1* silencing, as evidenced by the following: (1) the enrichment of RbAp46/48 was observed in the lncRNA-3-overexpressed mouse muscle satellite cells co-transfected with the full-length *MyoD1* promoter instead of the deletion fragments of the *MyoD1* promoter; (2) the *MyoD1* promoter was more significantly enriched in the lncRNA-3 overexpressed mouse muscle satellite cells than in the empty vector transfected cells according to ChIP analysis using anti-RbAp46/48 antibody; and (3) the blockage of the interaction between lncRNA-3 with PRC2 and the *MyoD1* promoter could promote *MyoD1* expression and elevate muscle regenerative capacity, respectively, in vitro and in vivo.

LncRNAs are appealing therapeutic targets because of the involvement in disease development, greater tissue specificity than protein-coding genes and multiple molecular mechanisms of regulating miRNAs or proteins [25]. However, there has still been no lncRNA-based drug brought into clinical trials to date due to the poor conservation of lncRNA cross-species [26]. Another challenge for lncRNA-based therapy is the difficulty in the modulation of lncRNAs and the potential systematic side effect [27]. In the current study, muscle-specific lncRNA-3 knockdown could restore the muscle-regenerative capacity in aged mice. Moreover, the exogenous RbAp46/48 binding motif treatment in skeletal muscle could compete for lncRNA-3 binding and inhibit the interaction between lncRNA-3 and PRC2, which, in turn, enhanced the muscle-regenerative capacity in the aged mice, implying that the interaction between lncRNA and its target gene/protein could be a promising therapeutic target.

We previously identified an lncRNA in mechanical-unloading-induced skeletal muscle atrophy (lncMUMA) [28], whose expression level in skeletal muscle was not altered during aging. We also identified an age-related lncRNA (MAR1) previously [29], which was a muscle anabolic enhancer. In contrast, in the current study, lncRNA-3 functioned as a muscle anabolic suppresser. The new understandings of the functional role of lncRNA-3 in skeletal muscle could help to complete the whole picture of anabolic and anti-anabolic mechanisms underlying muscle homeostasis during aging.

## 4. Methods

### 4.1. Animals

Adult and aged C57BL/6J mice were used in this study. All the animals were maintained under standard animal housing conditions (12 h light, 12 h dark cycles and free access to food and water). All the experimental procedures were approved by the Committees of Animal Ethics and Experimental Safety of the Chinese University of Hong Kong (AEEC no. 20-026-GRF).

### 4.2. Muscle Satellite Cells Isolation

The gastrocnemius muscles were minced for 7 min and digested with 0.1% (*w*/*v*) collagenase type II (Thermo Fisher Scientific, Waltham, MA, USA) and 2.5 units/mL Dispase II (Thermo Fisher Scientific, Waltham, MA, USA) in DMEM for 1 h with gentle trituration every 15 min. The digestion was terminated by the addition of 20% FBS in DMEM and processed for magnetic-activated cell sorting (MACS) analysis. The samples were passed through 100 μm and 70 μm filters. The cells were subjected to a red blood cell lysis and were incubated in MACS Buffer (0.5% BSA, 2 mM EDTA in PBS). The samples were subsequently incubated with antibodies conjugated to magnetic microbeads. The cells were incubated in anti-CD31 microbeads (1:10, Miltenyi, Bergisch Gladbach, Germany) and anti-C45 microbeads (1:20, Miltenyi, Bergisch Gladbach, Germany) for 15 min at 4 °C. The samples were washed and run through 70 µm filters into Miltenyi LS columns in the presence of a magnetic field. The flow-through was captured and then incubated in anti-α7-integrin microbeads (1:5, Miltenyi, Bergisch Gladbach, Germany) for 15 min at 4 °C and run through Miltenyi MS columns in the presence of a magnetic field. The columns were removed from the field and flushed to collect the CD31-/CD45-/α7-integrin+-enriched cells. The cells were washed and prepared for further culture.

### 4.3. Cell Culture

The freshly isolated satellite cells were plated in GM (20% FBS, 2.5 ng/mL basic-FGF, and 1× penicillin/streptomycin in DMEM) on 35 mm tissue culture plates coated with 1 mg/mL Matrigel (Corning, Corning, NY, USA). The cells were maintained in GM until they reached approximately 70% confluency (5–6 days). The cells were then switched to DM (10% horse serum and 1× penicillin/streptomycin in DMEM) and fed every 3 days. The cultured cells were fixed for 5 min in 4% PFA/PBS after 7 days in DM, rinsed in PBS and PBS-T (0.1% Triton X-100 in PBS), and processed for further analysis.

### 4.4. Skeletal Muscle Injury Animal Model

The skeletal muscle injury model was induced by an intramuscular injection with 50 μL of cardiotoxin (CTX) (10 μM) in the left gastrocnemius muscle of each mouse one day after the AAV particle injection [30].

### 4.5. Isolation of Total RNA and Real-Time PCR Analysis

The total RNA from the cell or muscle samples was isolated by a Trizol reagent (Invitrogen, Waltham, MA, USA) following the manufacturer’s instructions. cDNA syntheses for the mRNA and lncRNA detection were carried out using the SuperScript III first strand synthesis system for RT-PCR (Invitrogen, Waltham, MA, USA). The Fast Start Universal SYBR Green Master (Merck, Darmstadt, Germany) were applied for the quantitative real-time PCR. GAPDH and U6 were used as endogenous controls for normalization. All the primer sequences were listed in Supplementary Table S3. The relative fold changes of the candidate genes were analyzed by using the 2^−ΔΔCT^ method.

### 4.6. RNA Sequencing

RNA from 6 skeletal muscles from 3 adult and 3 aged mice were utilized for the RNA sequencing. The RNA sequencing was performed by the Illumina HiSeq 2000 platform (Illumina, San Diego, CA, USA). The RNA sequencing dataset was visualized by using the Integrative Genomics Viewer (IGV 2.8.9). Differentially expressed lncRNAs were identified through fold-change filtering (fold change ≥2.0 or ≤0.5), paired *t*-test (*p* < 0.05) and multiple hypothesis testing (FDR < 0.05). *p*-values and the FDR were calculated by Microsoft Excel 2016 and MATLAB 9.9, respectively.

### 4.7. Adeno-Associated Virus (AAV) Production and Cell Infection

For the construction of lncRNA-3 overexpression and shRNA AAV vector, full-length lncRNA-3 or specific shRNA sequences of lncRNA-3, together with mouse α-skeletal actin (MSA) promoter, were subcloned into the adeno-associated virus vector provided by Genechem Ltd. (Shanghai, China) according to the manufacturer’s instruction. For the cell infection, the cells were seeded into 6-well plates, and the AAVs (1.5 × 10^8^ IU per well) were added into the culture mediums and incubated for whole period [31].

### 4.8. Generation of Sk-lncRNA-3 KI Mice

To generate sk-lncRNA-3 KI mice, ROSA26-PCAG-STOP^flox^ –lncRNA-3 KI mice that carried the STOP^flox^-lncRNA-3 in the ROSA26 allele were successfully generated. In brief, a cassette that contained the following components was constructed to target the ROSA26 locus: FRT–LoxP–stop codons–three SV40 poly(A)–LoxP–lncRNA-3–WPRE–bGH poly(A)–AttB–promoter–FRT–Neo-PGK poly(A)–AttP. The targeting vector was constructed, fully sequenced and electroporated into C57BL/6 embryonic stem cells. Positive targeting clones were identified by PCR and Southern blotting. The targeted embryonic stem clones were microinjected into BALB/c blastocysts to obtain the chimeric mice. The chimeric mice were intercrossed with C57BL/6 mice to obtain the F1 heterozygote mice and then backcrossed with C57BL/6 mice to expand the number of ROSA26-PCAG-STOP^flox^ –lncRNA-3 KI mice. Then, these mice were intercrossed with ACTA1-Cre mice to obtain the Sk-lncRNA-3 KI mice. The littermates were used as the WT control.

### 4.9. In Vivo AAV Particle Administration and Peptide Treatment

The animals were anesthetized with an isophorone gas inhale system. For the AAV particle administration, 15 μL of viral preparation with a titer of 10.0 × 10^8^ IU/mL was injected into the mid-portion of the gastrocnemius muscles by using a Hamilton syringe (Merck, Darmstadt, Germany) with a 33G needle [28]. For the peptide treatment, 2 µg Rb-motif-2 in 40 µL saline was intramuscularly injected into the gastrocnemius muscle every two weeks for two months [32].

### 4.10. Tagged-RNA (DNA) Pulldown

To identify the direct binding partners of lncRNA-3, we used a tagged-RNA pulldown assay, as previously described [33]. Sense and antisense strands of streptavidin-binding S1m DNA were synthesized by Life Technologies Inc. (Carlsbad, CA, USA), annealed and cloned into pcDNA5-CMV in front of a cloning site, into which was inserted sequences that encoded wild-type or truncated versions of lncRNA-3; truncations were made at roughly 500 bp intervals from either the 5′ or 3′ end. These constructs, as well as those that expressed the untagged wild-type lncRNA-3 and EGFP, were transfected into the mouse muscle satellite cells for 48 h. The cells were then harvested in an SA-RNP lysis buffer (20 mM Tris-HCl (pH 7.5), 150 mM NaCl, 1.5 mM MgCl_2_, 2 mM DTT, 50 U/mL RNase OUT (Life Technologies, Carlsbad, CA, USA), 50 U/mL Superase IN (Ambion, Austin, TX, USA) and 1× complete protease inhibitor tablet (Merck, Darmstadt, Germany)). Streptavidin–sepharose beads were blocked with 500 ng/μL yeast tRNA and 1 mg/mL BSA in an SA-RNP lysis buffer before being added into cell lysates and incubated at 37 °C for 2 h on a rotator. The beads were then pelleted and washed five times with an SA-RNP washing buffer (20 mM Tris-HCl (pH 7.5), 300 mM NaCl, 5 mM MgCl_2_, 2 mM DTT, 50 U/mL RNase OUT (Life Technologies), 50 U/mL Superase IN and 1× complete protease inhibitor tablet). After the last wash, RNA-bound proteins were eluted by the addition of 5% RNase A (New England BioLabs, Ipswich, MA, USA) in a low-salt buffer (20 mM Tris-HCl (pH 7.5), 30 mM NaCl, 5 mM MgCl_2_, 2 mM DTT and 1× complete protease inhibitor tablet) for 30 min at 4 °C. The eluted proteins were then boiled in a 4× LDS sample buffer (Life Technologies, Carlsbad, CA, USA) and used for mass spectrometry (Agilent Technologies, Santa Clara, CA, USA) or Western blotting analysis.

### 4.11. Western Blotting

According to previously established procedures [34], the cell or muscle samples were harvested, washed with 1× PBS and lysed in NP40 lysis buffer (50 mM Tris-HCl, 150 mM NaCl, 0.1% NP-40, 5 mM EDTA, 10% glycerol) with a protease inhibitors cocktail (Sigma, Burlington, MA, USA). The samples were centrifuged and the supernatant was collected in a fresh tube. The proteins were separated in an SDS-PAGE, transferred and immunoblotted with various antibodies. The antibodies used were anti-MyoD (dilution 1:1000; Invitrogen), anti-RbAP46/48 (dilution 1:1000, Santa Cruz Biotechnology, Dallas, TX, USA) and anti-β-actin (dilution 1:3000; Santa Cruz Biotechnology, Dallas, TX, USA).

### 4.12. RNA Electrophoretic Mobility Shift Assay (EMSA)

An EMSA was performed essentially as described [35]. RNA probes were synthesized from a linearized pBluescript-lncRNA-3-66-mer and pBluescript-Hotair-89-mer using RiboMAX Large-Scale RNA Production Systems (Promega, Madison, WI, USA) and labeled with biotin using the RNA 3′ End Biotinylation Kit (Thermo Fisher Scientific, Waltham, MA, USA) following the manufacturer’s instructions. Recombinant RbAP46/48 was expressed from a linearized pT7CFE1- RbAP46/48 (Addgene, Watertown, MA, USA) in a coupled transcription and translation system (1-Step Human In Vitro Protein Expression Kits, Thermo Fisher Scientific, Waltham, MA, USA) following the manufacturer’s instructions. RNA EMSA was performed by using the LightShift Chemiluminescent RNA EMSA Kit (Thermo Fisher Scientific, Waltham, MA, USA). For each reaction, 10 pmol of labeled probe was incubated with 1 μL out of 20 μL in vitro translated RbAP46/48 in the presence of tRNA (1 mg/mL) at room temperature for 30 min. Unlabeled probes at the indicated concentrations were used for competition experiments. The reactions were then loaded onto a 1% 0.5 × TBE–agarose gel and transferred to a positively charged nylon membrane (Roche). The membrane was then cross-linked by exposure to UV, incubated with HRP-conjugated streptavidin and visualized with ECL reagents. The dissociation constant (Kd) was calculated as the concentration of the unlabeled probe when half of the labeled probe was dissociated from the complex with RbAP46/48.

### 4.13. Bioinformatics Analysis

The binding motifs between lncRNA-3 and RbAp46/48 and the potential target genes of lncRNA-3 were predicted by CatRAPID Omics (http://s.tartaglialab.com/page/catrapid_omics_group (accessed on 21 January 2021)). The Support Vector Machine (SVM) classifier was used to predict whether an atom within a molecule could be an SOM of FMO enzymes or not. An SVM can classify complex, non-linear and high-dimensional data into two classes. The merit of SVMs is they can classify data by mapping input vectors into a high- or infinite-dimensional space with some kernel functions and then construct a hyperplane or set of hyperplanes to separate them into two classes with a possible maximal margin computed. The margin is defined as the distance from the separating hyperplane to the nearest training data point. The trained model of an SVM classifier can be used to predict which class an unknown sample belongs to.

### 4.14. Tissue Preparation and Histological Analysis

The gastrocnemius muscles were collected and snap-frozen in liquid-nitrogen-cooled isopentane. The muscles were embedded in OCT medium, and 6 μm thick cross-sections were cut from the mid-belly of the muscles on a cryostat at −20 °C. Hematoxylin and eosin (H&E) staining was performed to determine the mean cross-sectional area (CSA) of the muscle fiber.

### 4.15. Immunohistochemistry

The sections were fixed in cold acetone (4 °C) for 20 min and then incubated in a 1% bovine serum albumin (BSA)/PBS solution for 1 h. The primary antibody against Pax7 was incubated overnight at 4 °C. A cocktail of secondary antibodies was incubated for 1 h at 37 °C. Sections were mounted in the Mowiol mounting medium (Merck, Darmstadt, Germany). The sections were visualized with an Axio Observer Z1 microscope (ZEN Lite, Carl Zeiss, Jena, Germany) using conventional widefield fluorescence microscopy, as well as optical sectioning via structured-illumination fluorescence microscopy (Apotome, Carl Zeiss, Jena, Germany). The Pax7+ cells were counted as satellite cell numbers per field by ImageJ (v1.54g) software. The measurements and counts made from histological sections were made by three independent observations of 5 random fields from a single observer.

### 4.16. EdU Staining

To study the satellite cell proliferation, the animals were injected intraperitoneally with a solution (50 mg/kg) of 5-ethynyl-2’-deoxyuridine (EdU) (Invitrogen) 4 h before being sacrificed. EdU staining was revealed according to the manufacturer guidelines. The sections were stained with 10 μM Alexa594-azide for 10–30 min. The sections were mounted in Mowiol mounting medium (Merck, Darmstadt, Germany) and observed under fluorescence microscopy. The proportions of EdU-positive cells in 5 random fields were calculated.

### 4.17. Statistical Analysis

Data are presented as the mean ± SEM from at least three independent experiments. Statistical differences between groups were analyzed by one-way analysis of variance (ANOVA) with a post hoc test for multiple comparisons of differences in the study parameters. All statistical analyses were performed with SPSS software version 22.0 (SPSS Inc., Chicago, IL, USA). *p* < 0.05 was considered statistically significant.

## 5. Conclusions

Taken together, the newly identified lncRNA-3 functions as an epigenetic regulator that recruits PRC2 to the *MyoD1* promoter for *MyoD1* silencing to suppress the muscle-regenerative capacity during aging. These findings provide a novel therapeutic target and a new strategy to elevate muscle regenerative capacity in the aged population.

## Data Availability

Data is contained within the article and Supplementary Materials.

## Supplementary Materials

The following supporting information can be downloaded from https://www.mdpi.com/article/10.3390/ijms252212478/s1.
