# Supplementary material for: Long Noncoding RNA lncRNA-3 Recruits PRC2 for MyoD1 Silencing to Suppress Muscle Regeneration During Aging
Source: Int J Mol Sci. 2024 Nov 20;25(22):12478. doi: 10.3390/ijms252212478 (PMC11594582; doi:10.3390/ijms252212478)
Supplement: Supplementary file 1 [file ijms-25-12478-s001.zip › ijms-3277876-supplementary.pdf]

Supplementary Table S1 List of top 10 up-regulated lncRNAs

lncRNA No.	Gene symbol	Fold change	P value
**1**	NR_108091.1	10.653554	1.68469E-05
**2**	XLOC_016124	7.7009767	9.16937E-08
**3**	XLOC_003036	5.6160685	0.000369922
**4**	ENSMUSG00000086841	4.5585821	0.00376057
**5**	XLOC_026701	4.4629132	0.003318087
**6**	XLOC_012529	4.1154752	0.000505835
**7**	ENSMUSG00000092267	3.9285529	1.12773E-05
**8**	ENSMUSG00000086320	3.6835207	0.000606465
**9**	ENSMUSG00000097624	3.6588797	0.002678243
**10**	ENSMUSG00000097589	3.2791555	0.000406768

Supplementary Table S2 Top 20 protein targets of lncRNA-3

Rank	Protein	Rank	Protein
1	RbAp46/48	11	HNRNPR
2	A2BP1	12	IGF2BP1
3	CPEB3	13	IGF2BP2
4	CSDA	14	KHDRBS1
5	EIF2S1	15	MATR3
6	EIF4B	16	NCL
7	HNRNPA1	17	PPRC1
8	YBX2	18	RBFOX2
9	HNRNPA3	19	RBM47
10	SRSF4	20	SNRPA

Supplementary Table S3 Primer sequences of lncRNA and mRNA

Gene	Primer sequences
lncRNA-3	forward: TGGCTTTAGTCCAGGCGAAG<br>reverse: TCATTGTCCGCAGCTTAGCA
Myod1	forward: AGTGAATGAGGCCTTCGAGA<br>reverse: GCATCTGAGTCGCCACTGTA

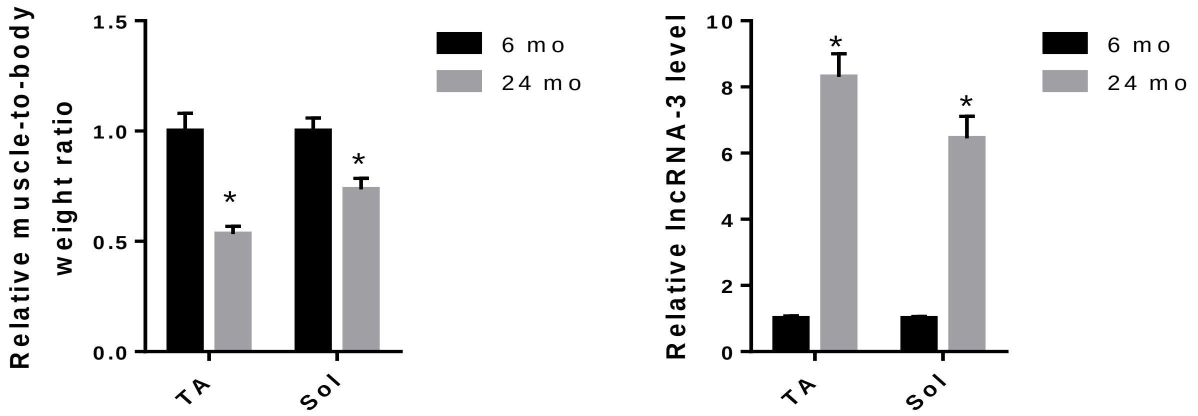

Supplementary Figure S1 Muscle weight and lncRNA-3 expresstion in TA and Sol between adult and aged mice. Skeletal muscle weight and expression level of lncRNA-3 in tibialis anterior (TA) and soleus (Sol) muscle of adult (6 mo) and aged (24 mo) mice. Data are presented as mean ± SEM. * *P*<0.05 vs. 6 mo.

>lncRNA-3 (Mouse)

AGCTTATTTCCAGCTGGCGAAGGGAAGAGCCTGGTTTCTCCAGTTTTTTTCTCCTCGGGGA
GTGCGCTGGAAGGGCGTGCTCCAGGCTTCCAGTTGGCTTTAGTCCAGGCGAAGCAGAAC
GCTCAGGCATCGAGTCCTCCAAGCTCACAGCTGCTTGGGCCGCGGCGGCGCCCCCACCA
CCCCCGGGTTTCTAGAGCCTTGTGACAGCGCCTCACTCCGAGCTGCTCAGCGGCTTTCTG
CAACTCTCTCTGCCTCGCCCCCAGTATGTGACGTGGGTGACAATGGCCCAGGTTGGAGCG
AGCCCCACGTCGGCGCGTCCTGGGTGGTCGGACCCGGGCAAACACAAATACAAACCGAT
TGCTAAGCTGCGGACAATGAGGGAAATGTAGACAAATGTCCCGCTCCTGTTGGAAGCCTTT
GTCCAGGCCCGGTTTTTGCATTTATTTCAGTGGCGAAATAATACATGATTGACGCTCTCTTT
CAATGTGTCCTAACTGTTTGGAATAAATCTAAGGTTGTCCCTAGTTGTCATGGCATTCAACC
CTTTTCAAAAAAAAAAAAAAAAAAA

Supplementary Figure S2 Sequence of mouse lncRNA-3.

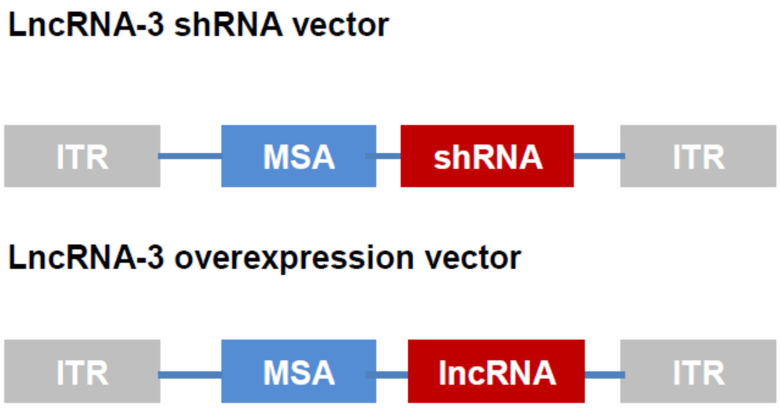

Supplementary Figure S3 Schematic of adeno-associated virus (AAV) vector used for overexpression or knockdown of lncRNA-3.

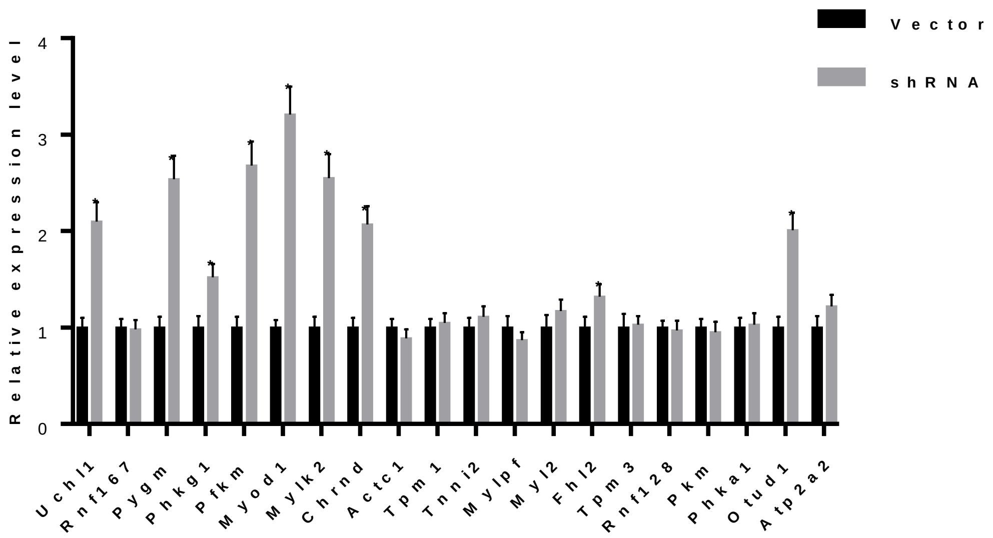

Supplementary Figure S4 Validation of MyoD1 as a target gene of lncRNA-3. Real-time PCR analysis of top 20 predicted target genes of lncRNA-3 in lncRNA-3-shRNA or empty vector transduced mouse muscle satellite cells. Data are presented as mean ± SEM. * *P*<0.05 vs. vector control.
